# Platelet-to-Lymphocyte Ratio as a Predictor of Lymphovascular Space Invasion in Endometrioid Endometrial Cancer: Development and Internal Validation of a Continuous Parameter-Based Nomogram

**DOI:** 10.3390/medicina62061190

**Published:** 2026-06-19

**Authors:** Kasim Akay, Gorkem Ulger, Hamza Yildiz, Zeynep Kucukolcay Coskun, Sevki Goksun Gokulu, Tolgay Tuyan Ilhan, Hakan Aytan

**Affiliations:** 1 Department of Obstetrics and Gynecology, Osmaniye Düziçi State Hospital, 80600 Osmaniye, Turkey; 2 Department of Obstetrics and Gynecology, Faculty of Medicine, Mersin University, 33110 Mersin, Turkey; gorkem.ulger@gmail.com (G.U.); drzkucukolcay@gmail.com (Z.K.C.); sevkigoksungokulu@gmail.com (S.G.G.); tolgaytuyan@yahoo.com (T.T.I.); drhakanaytan@yahoo.com (H.A.); 3 Department of Obstetrics and Gynecology, Mersin Tarsus State Hospital, 33400 Mersin, Turkey; hamzayldz911@gmail.com

**Keywords:** platelet-to-lymphocyte ratio, systemic inflammation, endometrial cancer, lymphovascular space invasion, nomogram, predictive model, bootstrap validation

## Abstract

*Background and Objectives*: The relationship between preoperative inflammatory markers and lymphovascular space invasion (LVSI) in endometrioid-type endometrial cancer (EC) remains incompletely defined and warrants evaluation using robust statistical methods. This study aimed to evaluate the independent association of preoperative inflammatory markers, analyzed strictly as continuous variables, with the presence of LVSI, and to develop a refined predictive nomogram adjusted for established clinical confounders. *Materials and Methods*: Data from 156 patients who underwent standard staging surgery for endometrioid-type EC were retrospectively analysed. To preserve statistical power and avoid structural artifacts from data forcing, preoperative glucose-to-lymphocyte ratio (GLR), platelet-to-lymphocyte ratio (PLR), and neutrophil-to-lymphocyte ratio (NLR) were modeled on their original continuous scale. Multivariable logistic regression analysis was performed to identify independent risk factors for LVSI, adjusting for patient age and maximum tumor diameter. Internal validation was conducted using bootstrap resampling (1000 iterations). *Results*: In the multivariable logistic regression model, continuous PLR emerged as a significant independent risk factor for the presence of LVSI (adjusted OR: 1.013 per 1-unit increase, 95% CI: 1.001–1.024; *p* = 0.033). Among clinical parameters, maximum tumor diameter demonstrated the strongest independent association with LVSI (adjusted OR: 1.595 per 1 cm increase, 95% CI: 1.211–2.099; *p* = 0.001). Continuous NLR (*p* = 0.513) and GLR (*p* = 0.545) did not retain statistical significance due to overlapping explanatory variance and shared hematological components. The optimized 3-variable nomogram (PLR, tumor size, and age) demonstrated an apparent C-index of 0.816 (95% bootstrap CI: 0.719–0.920) and a robust optimism-corrected C-index of 0.794. The bootstrap-corrected calibration slope was 0.909, and Decision Curve Analysis (DCA) demonstrated a positive net clinical benefit across clinically relevant threshold probabilities. *Conclusions*: Preoperative PLR, evaluated as a continuous parameter, provides a statistically stable framework for preoperative risk stratification in endometrioid EC. When integrated with tumor size and age, the proposed nomogram demonstrates promising discriminative performance and potential clinical utility pending external validation for predicting LVSI. However, given the limited number of LVSI-positive events (*n* = 17), these findings should be regarded as exploratory and hypothesis-generating and require external validation before clinical use.

## 1. Introduction

Endometrial cancer (EC) ranks among the most common gynecological malignancies affecting women in developed countries, with its incidence steadily increasing [1,2]. Endometrioid-type EC constitutes approximately 75–80% of all endometrial cancers and generally has a better prognosis; however, its heterogeneous nature complicates prognostic assessment and treatment planning [3,4].

In recent years, the critical role of systemic inflammation in the tumor microenvironment and its impact on cancer prognosis has been an area of growing investigation [5,6]. The inflammatory response contributes to tumorigenesis by causing DNA damage, stimulating angiogenesis, and potentiating pro-proliferative and anti-apoptotic processes [7]. In this context, systemic inflammatory markers such as neutrophil to lymphocyte ratio (NLR), platelet to lymphocyte ratio (PLR), and Systemic Immune-Inflammation Index (SII) have emerged as biomarkers with prognostic value in various cancer types [8,9]. Recently, new inflammatory indices including the glucose-to-lymphocyte ratio (GLR) have shown promising results in cancer prognosis [10,11].

One of the most significant innovations of the 2023 FIGO staging system is the more prominent inclusion of lymphovascular space invasion (LVSI) assessment in the system [12,13]. LVSI, characterized by tumor cell invasion into blood and lymphatic vessels, is recognized as a prognostic indicator closely associated with tumor aggressiveness, lymph node metastasis risk, and recurrence rates [14,15]. The more detailed evaluation of LVSI in the 2023 FIGO staging system indicates that this parameter has evolved from being merely a prognostic indicator to a factor directly influencing staging and treatment decisions. This development underscores the growing clinical need for accurate preoperative prediction of LVSI.

The primary aim of this study was to evaluate the predictive value of preoperative inflammatory markers (GLR, PLR, NLR, and SII) for the presence of LVSI in endometrioid-type EC by treating them as unforced, continuous variables to preserve statistical integrity. The secondary aim was to develop and internally validate a refined preoperative nomogram that balances systemic inflammatory-metabolic parameters with fundamental clinicopathological confounders, thereby providing a methodologically stable tool for individualized risk estimation.

## 2. Materials and Methods

This retrospective cohort study encompasses patients who underwent surgical treatment for endometrioid-type EC at Mersin University Hospital Department of Obstetrics and Gynecology between January 2019 and December 2023. Approval was obtained from Mersin University Ethics Committee for the study (Approval number: 52, Date: 6 March 2024). This study was performed in line with the principles of the Declaration of Helsinki. Patient consent was waived due to the retrospective nature of the study. All data were obtained from existing medical records and analysed anonymously.

**Figure 1:**
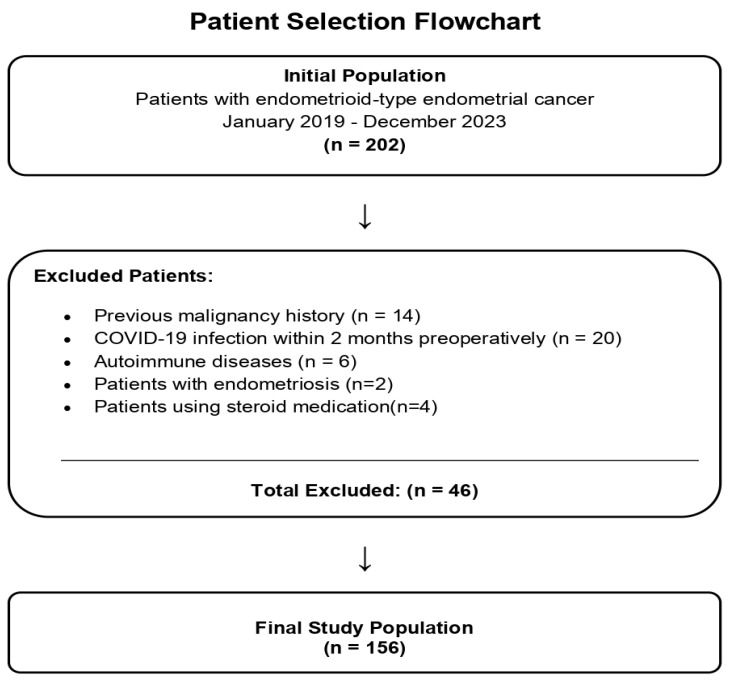
Patient Selection Flowchart.

Inclusion criteria were defined as: patients aged 18 years and older, operated for endometrioid-type EC, and who had not previously undergone surgical intervention for EC. Exclusion criteria included: patients previously operated for EC, patients with non-endometrioid type EC, patients with a history of chemotherapy or radiotherapy, patients with insufficient medical records, patients under 18 years of age, patients with a previous history of malignancy (*n* = 14), patients with autoimmune disease (*n* = 6), patients with endometriosis (*n* = 2), patients using steroid medication (*n* = 4), and patients who had COVID-19 disease within the preoperative 2-month period (*n* = 20). The 2-month exclusion threshold for prior COVID-19 infection was applied conservatively, as evidence indicates that lymphocyte counts and NLR normalize rapidly during convalescence, reaching levels comparable to healthy controls within weeks of symptom resolution [16]. Of the 202 patients initially evaluated, 156 were included in the final analysis (Figure 1). Exclusion criteria were applied hierarchically; no patient met more than one exclusion criterion.

Standard staging surgery was performed on all patients. The basic surgical procedure included total abdominal hysterectomy + bilateral salpingo-oophorectomy (TAH + BSO) or laparoscopic hysterectomy + bilateral salpingo-oophorectomy. Peritoneal cytology samples were routinely obtained from all patients. Tumor size, degree of myometrial invasion, and tumor grade were evaluated with intraoperative frozen section analysis. Pelvic lymphadenectomy was performed in all patients as part of standard surgical staging. In patients whose tumor diameter exceeded 2 cm on preoperative imaging, pelvic lymphadenectomy was performed without awaiting intraoperative frozen section results, whereas in patients with smaller tumors the procedure was performed following frozen section evaluation. Pelvic-paraaortic lymphadenectomy was additionally performed in patients identified as high-risk according to intraoperative frozen section findings.

Patients’ demographic characteristics, gravidity, parity numbers, comorbidities, and malignancy histories were recorded. Complete blood count (hemoglobin, hematocrit, leukocyte, neutrophil, lymphocyte, platelet, monocyte counts), fasting blood glucose, and serum CA-125 levels were obtained from routine laboratory tests taken in the preoperative period within 7 days prior to surgery (median 1 day, range 1–6 days). All preoperative blood tests were performed at the central laboratory of Mersin University Faculty of Medicine using a standardized automated hematology analyzer (SYSMEX XN-1000, Sysmex Corporation, Kobe, Japan). Inflammatory markers were calculated from preoperative laboratory values: Platelet to Lymphocyte Ratio (PLR): Platelet count/Lymphocyte count, Neutrophil to Lymphocyte Ratio (NLR): Neutrophil count/Lymphocyte count, Systemic Immune-Inflammation Index (SII): (Platelet count × Neutrophil count)/Lymphocyte count, Glucose to Lymphocyte Ratio (GLR): Fasting blood glucose/Lymphocyte count.

Data obtained from postoperative pathology examination included: tumor type and size, histological grade (Grade 1, 2, 3 according to FIGO system), presence of lymphovascular space invasion (LVSI), presence and degree of myometrial invasion, presence of cervical stromal invasion, lymph node metastasis status, presence of distant organ metastasis, and FIGO staging. LVSI assessment was performed only on final hysterectomy specimens due to limited tissue sampling and potential sampling error found in preoperative biopsies. LVSI was recorded as a binary variable (present vs. absent) according to standard pathological evaluation criteria. The distinction between focal and substantial LVSI, as defined by the 2023 FIGO staging system, was not applied in this study, which represents a limitation.

Statistical analyses were performed using IBM SPSS version 26.0 (IBM Corp., Armonk, NY, USA) and MedCalc Statistical Software version 20.211 (MedCalc Software, Ostend, Belgium) for univariable receiver operating characteristic (ROC) curves. The predictive nomogram and its subsequent validation steps were constructed using the R programming language (version 4.4.0; R Foundation for Statistical Computing, Vienna, Austria) via the RStudio interface (version 2024.04.2+764; Posit Software, PBC, Boston, MA, USA) with the "rms" package (version 6.8; Harrell FE Jr., Vanderbilt University, Nashville, TN, USA). The normality of data distribution was assessed using the Shapiro–Wilk test. Continuous variables with normal distribution were presented as mean ± standard deviation, non-normally distributed continuous variables as median (interquartile range, Q1–Q3), and categorical variables as frequencies and percentages. Group comparisons were performed using Student’s *t*-test, the Mann–Whitney U test, or the Chi-square/Fisher’s exact test, as appropriate.

To preserve statistical power, maintain clinical reproducibility, and prevent artificial artifacts arising from data forcing, all candidate predictors were modeled on their original continuous scale. To ensure the statistical independence of the prediction model and explicitly avoid structural redundancy or multicollinearity among inflammatory ratios derived from overlapping hematological components (specifically the shared lymphocyte denominator), only one primary inflammatory index—the Platelet-to-Lymphocyte Ratio (PLR)—was entered into the multivariable framework alongside pre-specified clinical confounders. Independent risk factors for the presence of LVSI were evaluated using multivariable binary logistic regression analysis (Enter method), incorporating continuous patient age (years), maximum tumor diameter (cm), and continuous PLR. Adjusted Odds ratios (OR) and 95% confidence intervals (CI) were calculated. Multicollinearity was rigorously assessed using individual Variance Inflation Factors (VIF).

A predictive clinical nomogram was constructed based on the optimized multivariable logistic regression coefficients. Internal validation of the nomogram was executed via bootstrap resampling with 1000 iterations to estimate and correct for potential in-sample overfitting. The apparent and optimism-corrected C-indices (Area Under the Curve, AUC) were calculated. Model calibration was comprehensively evaluated using a restricted cubic spline calibration curve accompanied by the bootstrap-corrected calibration slope and intercept, alongside the Hosmer-Lemeshow goodness-of-fit test and the overall Brier score. Finally, Decision Curve Analysis (DCA) was applied across a clinical range of threshold probabilities to quantify the net clinical benefit of the nomogram. Statistical significance was defined as *p* < 0.05.

It should be noted that with 17 LVSI-positive events and 3 predictor variables, the events-per-variable (EPV) ratio was approximately 5.7, which is below the commonly recommended minimum of 10 for logistic regression models. All multivariate results should therefore be interpreted as exploratory and hypothesis-generating. Post hoc power analysis was not performed, as it has been criticized for providing limited additional information beyond the *p*-value itself [17]. With an LVSI positivity rate of 10.9% (17/156), the sample was sufficient for primary comparisons at α = 0.05.

## 3. Results

A total of 156 patients with endometrioid-type EC were included in the study. The basic demographic and clinical characteristics of patients are presented in Table 1. The mean age of patients was 60.01 ± 9.31 years. Of the patients, 118 (75.6%) were 55 years and older, while 38 (24.4%) were under 55 years. All patients underwent pelvic lymphadenectomy, and 63 (40.4%) additionally underwent pelvic-paraaortic lymphadenectomy based on intraoperative frozen section findings. LVSI status was assessed from the final hysterectomy specimen in all 156 patients, independent of lymphadenectomy extent.

When demographic parameters were compared between LVSI-positive and LVSI-negative patient groups, the mean age was 58.06 ± 10.23 years in LVSI-positive patients and 60.24 ± 9.21 years in LVSI-negative patients (*p* = 0.363). No significant difference was found between groups in terms of gravidity [3.00 (2.00–3.50) vs. 2.00 (1.00–4.00), *p* = 0.497] or parity [3.00 (2.00–3.50) vs. 2.00 (1.00–3.00), *p* = 0.187] (Table 1).

Tumor size was significantly larger in LVSI-positive patients [5.50 cm (4.00–7.00) vs. 3.00 cm (2.00–4.00), *p* < 0.001]. Hemoglobin value was significantly lower in LVSI-positive patients [12.00 g/dL (10.70–12.80) vs. 13.00 g/dL (12.20–14.00), *p* = 0.002], while platelet count was significantly higher [306.00 × 10^3^/µL (282.00–358.00) vs. 281.00 × 10^3^/µL (239.00–316.00), *p* = 0.036] (Table 1).

Serum CA-125 level was significantly higher in LVSI-positive patients [25.90 U/mL (20.52–51.05) vs. 15.20 U/mL (11.40–28.20), *p* = 0.030]. No significant difference was found between groups in terms of other laboratory parameters (leukocyte, neutrophil, lymphocyte, monocyte counts and glucose levels) (*p* > 0.05 for all) (Table 1).

### Development of Prediction Model for LVSI

All candidate predictors were modelled on their original continuous scale. Three pre-specified variables were considered for the prediction model: patient age (years), maximum tumor diameter (cm) and the platelet-to-lymphocyte ratio (PLR). To preserve the statistical independence of the predictors and to avoid redundancy among inflammatory ratios derived from overlapping haematological components, only one inflammatory index (PLR) was entered into the multivariable model. In univariable logistic regression, tumor size (unadjusted OR 1.695 per 1 cm increase, 95% CI 1.297–2.215; *p* < 0.001) and PLR (unadjusted OR 1.016 per 1-unit increase, 95% CI 1.006–1.027; *p* = 0.002) were significantly associated with LVSI, whereas age was not (unadjusted OR 0.975 per year, 95% CI 0.923–1.029; *p* = 0.361). Age was retained in the multivariable model as a pre-specified clinical confounder. In the multivariable logistic regression, both tumor size (adjusted OR 1.595 per cm, 95% CI 1.211–2.099; *p* = 0.001) and PLR (adjusted OR 1.013 per unit, 95% CI 1.001–1.024; *p* = 0.033) remained independently associated with LVSI after mutual adjustment, while age did not reach statistical significance (adjusted OR 0.968 per year, 95% CI 0.912–1.027; *p* = 0.286) (Table 2). The likelihood ratio test confirmed a strong overall model fit (χ^2^ = 22.59, df = 3, *p* < 0.001; Nagelkerke R^2^ = 0.271).

Multicollinearity was assessed prior to model interpretation. Pairwise Spearman correlations among the three predictors were negligible (|ρ| ≤ 0.17 for all pairs), and variance inflation factors derived from the multivariable model were all close to unity (age VIF = 1.00, tumor size VIF = 1.05, PLR VIF = 1.06), well below conventional thresholds and indicating the absence of structural collinearity.

**Figure 2:**
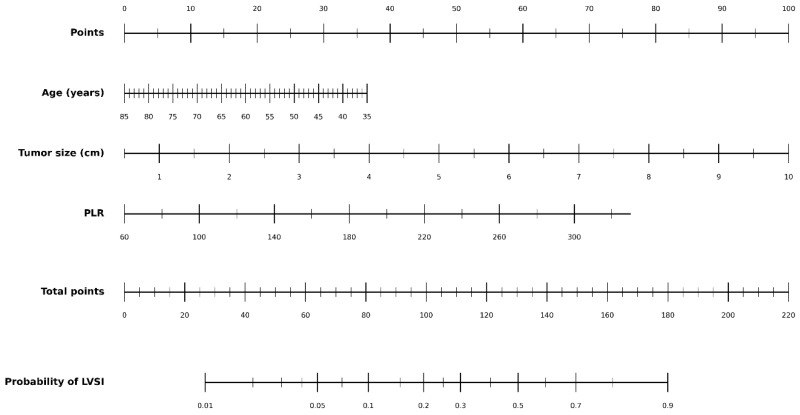
Nomogram for the preoperative prediction of lymphovascular space invasion (LVSI) in endometrioid endometrial cancer, based on continuous patient age, tumor size and platelet-to-lymphocyte ratio (PLR).

A prediction nomogram was constructed on the basis of the multivariable model, with each continuous predictor contributing a graphically determined number of points proportional to its regression coefficient and clinical range (Figure 2). The clinical range of each axis was set slightly beyond the observed range of the study cohort (age 35–85 years; tumor size 0.5–10 cm; PLR 60–330) to allow extrapolation to plausible values encountered in routine practice. The linear predictor underlying the nomogram is:

logit P(LVSI) = −4.013 − 0.0324 × Age + 0.4666 × Tumor size + 0.0125 × PLR

To use the nomogram in clinical practice, the value of each predictor is located on its corresponding axis, a vertical line is drawn upward to the “Points” axis to obtain the partial score for that variable, the three partial scores are summed to obtain the “Total points” score, and a vertical line is then projected downward from the total score to the “Probability of LVSI” axis to read the predicted probability. As a worked example, a 60-year-old woman with a 4 cm tumor and a preoperative PLR of 150 receives approximately 18 points for age, 37 points for tumor size and 25 points for PLR, yielding a total of 80 points, which corresponds to a predicted probability of LVSI of approximately 10%. A 55-year-old woman with a 6 cm tumor and a PLR of 200 would reach a total score of approximately 130 points, corresponding to a predicted probability of LVSI of approximately 38%.

**Figure 3:**
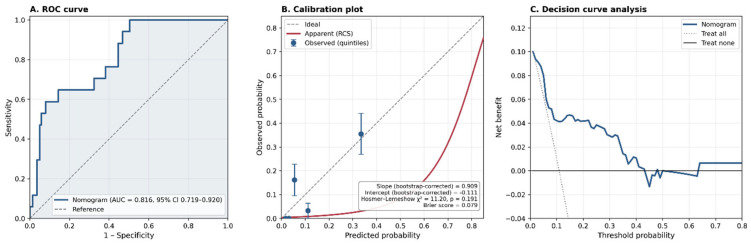
Internal validation of the LVSI prediction nomogram. (**A**) Receiver operating characteristic (ROC) curve with apparent AUC and bootstrap 95% confidence interval; the optimism-corrected AUC was 0.794. (**B**) Apparent calibration plot with restricted cubic spline calibration curve (red line) and observed event rates in quintiles of predicted probability (blue points with 95% confidence intervals); the dashed diagonal represents perfect calibration. The bootstrap-corrected calibration slope and intercept are inset. (**C**) Decision curve analysis showing the net benefit of the nomogram compared with the “treat all” and “treat none” reference strategies across a range of threshold probabilities.

Internal validation of the nomogram was performed using bootstrap resampling with 1000 iterations. The apparent area under the receiver operating characteristic curve (AUC) was 0.816 (95% bootstrap percentile CI 0.719–0.920), and the optimism-corrected AUC was 0.794, indicating good discrimination after correction for in-sample over-fitting (Figure 3A). The bootstrap-corrected calibration slope was 0.909 and the bootstrap-corrected calibration intercept was −0.111, both close to their ideal values of 1 and 0, respectively, corresponding to a calibration slope shrinkage factor of less than 10%. The restricted cubic spline calibration curve closely followed the diagonal across the observed range of predicted probabilities, and observed event rates by quintiles of predicted risk agreed with predicted probabilities within their 95% confidence intervals (Figure 3B). The Hosmer–Lemeshow test did not reject adequate model fit (χ^2^ = 11.20, df = 8, *p* = 0.191), and the overall Brier score was low (0.079). Decision curve analysis demonstrated that the nomogram yielded a higher net benefit than both the “treat all” and the “treat none” reference strategies across the clinically relevant threshold range of approximately 5–40% (Figure 3C), supporting its potential utility for preoperative risk stratification. At the Youden-optimal predicted-probability cut-off of 0.23, the nomogram achieved a sensitivity of 58.8%, a specificity of 92.1%, a positive predictive value of 47.6% and a negative predictive value of 94.8%.

With 17 LVSI-positive events and three continuous predictors, the events-per-variable ratio of the final model was 5.67. Predictors were pre-specified rather than selected by data-driven procedures; no collinearity was present, and internal validation by bootstrap with shrinkage assessment was performed. The narrow optimism observed for the AUC (ΔAUC = 0.022) and a calibration slope close to unity (0.909) provide direct empirical evidence that overfitting in the present model is limited. Given the small absolute number of events and the single-centre design, the proposed nomogram should be regarded as a hypothesis-generating tool requiring external, ideally multicentre, validation before clinical implementation.

## 4. Discussion

The present study suggests that preoperative platelet-to-lymphocyte ratio (PLR) is an independent predictor of lymphovascular space invasion (LVSI) in endometrioid-type EC. The nomogram incorporating PLR alongside tumor size and patient age shows promising discriminative performance for preoperative LVSI risk stratification. Our findings are broadly consistent with the current literature and additionally offer methodologically relevant contributions.

LVSI has evolved considerably within the 2023 FIGO staging system, transcending its prior role as a purely prognostic parameter to become a determinant that directly influences staging assignment and adjuvant treatment recommendations. As highlighted by Berek et al. in the FIGO 2023 update, the distinction between focal LVSI, classified within Stage IA2, and substantial LVSI, separately categorized as Stage IIB, has substantially elevated the clinical weight of this pathological feature [18]. A large multi-institutional retrospective study from Japan demonstrated that 36.3% of patients were reclassified upon re-staging under the FIGO 2023 system, with 46 patients (19.6%) being upstaged to an intermediate-risk category solely on the basis of focal LVSI. Notably, the presence of substantial LVSI was associated with a marked reduction in 3-year overall survival to 40.7% [19]. These data underscore the clinical imperative of accurately predicting LVSI status in the preoperative period to guide surgical planning and adjuvant therapy decisions.

The association between PLR and LVSI constitutes the principal finding of the present study. In a prospective multicenter study by Ronsini et al. enrolling 163 patients with Stage I endometrial cancer, NLR, MLR, and PLR were each found to correlate significantly with the degree of myometrial infiltration, LVSI positivity, and tumor grade; the median PLR in LVSI-positive cases was 141, compared with 125 in LVSI-negative cases (*p* = 0.033) [20]. These findings are consistent with the results of the present study, in which PLR was significantly elevated in LVSI-positive patients (168.25 vs. 123.96; *p* = 0.007) and retained independent predictive value in multivariable analysis (adjusted OR: 1.013 per unit increase; *p* = 0.033).

A distinguishing methodological feature of the present study is the modeling of PLR as a continuous variable rather than employing a predetermined dichotomous cutoff, as commonly applied in prior research. This approach carries important statistical advantages: the imposition of an arbitrary threshold results in loss of statistical power, compromises clinical reproducibility, and introduces structural artifacts into the prediction model. By contrast, continuous modeling allows the estimated effect of PLR on LVSI risk to be expressed as a mathematically unforced, unit-wise odds ratio (OR: 1.013), thereby providing a clinically transparent and statistically stable framework for individual risk estimation. It should be emphasized that although the per-unit odds ratio of PLR appears modest (OR: 1.013), this reflects the effect of a single-unit increase and should not be interpreted as clinically negligible. Because PLR varies across a wide range in clinical practice, its cumulative effect becomes substantially more meaningful over broader intervals; for instance, a per 50-unit increase in PLR corresponds to an approximately 1.9-fold increase in the odds of LVSI (1.013^50^ ≈ 1.91). This distinction is particularly relevant given the difference in median PLR between LVSI-positive and LVSI-negative patients in our cohort (168.25 vs. 123.96), and underscores that the unit-based odds ratio should be interpreted across clinically relevant ranges rather than in isolation.

The prognostic significance of PLR in endometrial cancer is well-established in the meta-analytic literature. The systematic review and meta-analysis by Leng et al., encompassing 14 studies and 5274 patients, demonstrated that elevated NLR and PLR were independent predictors of both overall survival and disease-free survival (multivariate OS HR for PLR: 1.86; 95% CI: 1.22–2.83) [21]. Ni et al. similarly reported that elevated PLR was associated with inferior overall survival (pooled HR: 1.99; 95% CI: 1.51–2.61) and progression-free survival (pooled HR: 2.02; 95% CI: 1.45–2.80) in endometrial cancer patients [22]. These meta-analytic findings support the clinical relevance of PLR in endometrial cancer. The present study extends this evidence by demonstrating that PLR is not only prognostically informative but also capable of predicting the preoperative presence of LVSI when modeled as a continuous variable.

The biological basis underlying the association between PLR and LVSI is grounded in platelet-tumor microenvironment interactions. As comprehensively reviewed by Rab et al., platelets promote tumor cell proliferation, migration, and invasion through the secretion of platelet-derived growth factor (PDGF), vascular endothelial growth factor (VEGF), and transforming growth factor-beta (TGF-β) [23]. These mediators establish a pro-angiogenic and pro-invasive microenvironment that facilitates tumor cell access to the lymphovascular space. Furthermore, platelets contribute to immune evasion by suppressing T-cell function and promoting the activity of myeloid-derived suppressor cells, thereby shielding circulating tumor cells from immune surveillance. These immunomodulatory mechanisms provide a plausible biological explanation for the observed association between elevated platelet counts, higher PLR values, and increased LVSI risk.

The identification of maximum tumor diameter as the strongest independent predictor of LVSI in the present cohort (adjusted OR: 1.595 per cm increase; *p* = 0.001) is consistent with the broader literature. In a series of 582 patients reported by Çaltek et al., a preoperative model incorporating multiple clinical variables demonstrated high predictive performance for LVSI, with CA-125 and tumor size emerging as important contributors (AUC: 0.886) [24]. Cao et al., in a study of 424 patients with low-grade endometrioid EC, similarly identified myometrial invasion depth and CA-125 as the strongest independent predictors of lymph node metastasis, with LVSI serving as a key intermediary of this association [25]. The significantly larger tumor size observed in LVSI-positive patients in our cohort (median 5.50 cm vs. 3.00 cm in LVSI-negative patients) is consistent with these findings.

Imaging-based approaches to the preoperative prediction of LVSI have gained considerable momentum in recent years. Yi et al., in a multicenter cohort of 580 patients, developed a comprehensive model (CRDL model) integrating MRI-based radiomic and deep learning features with clinical variables, achieving an AUC of 0.924 in the training cohort and 0.831 in the external validation cohort [26]. Yan et al. constructed an MRI-based radiomics nomogram incorporating both intratumoral and peritumoral regions in endometrioid adenocarcinoma, reporting AUC values of 0.962 and 0.965 in the training and validation cohorts, respectively [27]. Although these imaging-based models demonstrate superior discriminative accuracy, they necessitate advanced technical infrastructure and specialized expertise that may not be universally available. The principal practical advantage of the nomogram proposed in the present study lies in its reliance exclusively on routinely obtained preoperative complete blood count parameters, making it an accessible, cost-effective tool for preoperative LVSI risk stratification without requiring additional diagnostic workup.

The failure of NLR and GLR to reach statistical significance in the multivariable model of the present study is best attributed to the structural collinearity inherent among inflammatory ratios sharing a common denominator (lymphocyte count), which results in overlapping explanatory variance. This statistical phenomenon supports the decision to include only PLR as the primary inflammatory index in the multivariable framework. The study by Özbilgeç et al., in a series of 194 patients, reported that PLR was a significant predictor of advanced-stage disease but demonstrated limited association with LVSI specifically [28], illustrating how differences in cohort size, LVSI prevalence, and methodological design can yield divergent results across studies and warranting caution in cross-study comparisons.

The internal validation performance of the present nomogram, reflected by an optimism-corrected C-index of 0.794, adequate calibration on the Hosmer-Lemeshow test, and a positive net benefit over both treat-all and treat-none reference strategies on decision curve analysis, suggests potential clinical utility. These results are broadly comparable to those reported by Huang et al., whose SII-based nomogram for endometrial cancer achieved a C-index of 0.866, with postoperative SII identified as an independent prognostic factor [29]. A critical distinction, however, is that the nomogram presented here relies solely on preoperative parameters, enabling its direct integration into the preoperative surgical planning process rather than requiring postoperative data.

The quantitative assessment of LVSI extent has emerged as an area of active investigation. Sırma et al. recently proposed a three-tiered LVSI classification comprising focal (1–4 vessels), substantial (5–9 vessels), and prominent (≥10 vessels) categories, demonstrating that prominent LVSI was a powerful independent predictor of mortality (HR: 14.31; *p* < 0.001) [30]. In the large series of 2091 patients reported by Capasso et al., focal LVSI was not associated with significantly inferior survival outcomes compared with LVSI-negative cases, whereas substantial LVSI independently predicted reduced disease-free and overall survival, even within molecularly defined low-risk subgroups [31]. These findings collectively highlight that the binary classification of LVSI as present or absent may underestimate the prognostic heterogeneity within LVSI-positive tumors, and that quantitative assessment is essential for refined risk stratification. The inability to classify LVSI as focal or substantial in accordance with the 2023 FIGO staging criteria in the present study represents a notable limitation of its retrospective design, as this distinction directly influences adjuvant treatment recommendations.

Several limitations of the present study merit consideration. The single-center retrospective design and the limited number of LVSI-positive events (*n* = 17) constrain the statistical power and generalizability of the findings. The events-per-variable ratio of approximately 5.7 falls below the commonly recommended threshold of 10 for logistic regression. However, the pre-specification of predictors rather than data-driven selection, the absence of multicollinearity, and the small degree of optimism observed on bootstrap validation (ΔAUC = 0.022; calibration slope = 0.909) provide empirical evidence that overfitting in the present model is limited. Nonetheless, external validation in large-scale, multicenter, prospective cohorts is an essential prerequisite before clinical implementation. Two further limitations warrant explicit emphasis. First, the present model predicts only the binary status of LVSI (present vs. absent) and does not distinguish between focal and substantial LVSI, the latter distinction being directly incorporated into the 2023 FIGO staging system and into contemporary adjuvant treatment decisions; the predicted probability should therefore be interpreted as the likelihood of any LVSI rather than of a specific, clinically refined LVSI category. Second, molecular classification was not available in this cohort, and the model does not account for the prognostic information conveyed by molecular subtypes. Future studies should also incorporate molecular subtyping (POLE mutation, mismatch repair deficiency, p53-aberrant, and NSMP subtypes) alongside inflammatory markers to clarify their combined predictive value and underlying biological basis.

## 5. Conclusions

In conclusion, the present exploratory study suggests that preoperative PLR, evaluated as a continuous parameter, may be an independent predictor of LVSI in endometrioid-type EC. The three-variable nomogram integrating PLR with tumor size and patient age demonstrates promising discriminative performance and a net clinical benefit across a clinically relevant range of threshold probabilities. This nomogram may represent a preliminary tool for individualized LVSI risk estimation; however, external multicenter validation is mandatory before any clinical implementation.

## Figures and Tables

**Table 1:** Basic characteristics of the study population according to LVSI status.

Demographic Data and Laboratory Parameters	LVSI(−) *n* = 139	LVSI(+) *n* = 17	*p*-Value
Age (year)	60.24 ± 9.21	58.06 ± 10.23	0.363
Gravidity	2.00 (1.00–4.00)	3.00 (2.00–3.50)	0.497
Parity	2.00 (1.00–3.00)	3.00 (2.00–3.50)	0.187
Tumor size (cm)	3.00 (2.00–4.00)	5.50 (4.00–7.00)	<0.001
Leukocytes (×10^3^/µL)	8.38 ± 2.49	8.54 ± 2.41	0.808
Hemoglobin (g/dL)	13.00 (12.20–14.00)	12.00 (10.70–12.80)	0.002 *
Platelets (×10^3^/µL)	281.00 (239.00–316.00)	306.00 (282.00–358.00)	0.036 *
Neutrophil (×10^3^/µL)	5.38 ± 1.98	5.70 ± 1.75	0.455
Lymphocytes (×10^3^/µL)	2.17 (1.77–2.71)	2.22 (1.68–2.88)	0.566
Monocytes (×10^3^/µL)	0.55 (0.45–0.67)	0.56 (0.38–0.59)	0.324
Glucose (mg/dL)	107.60 (93.20–128.60)	116.00 (101.50–151.00)	0.096
CA-125 (U/mL)	15.20 (11.40–28.20)	25.90 (20.52–51.05)	0.030 *
PLR	123.96 (101.11–157.02)	168.25 (131.53–210.59)	0.007 *
NLR	2.28 (1.62–3.03)	2.54 (2.08–3.09)	0.163
SII	614.69 (455.68–919.53)	783.93 (650.83–1024.77)	0.012 *
GLR	48.90 (39.49–62.46)	53.12 (47.94–79.41)	0.137

Footnotes: Data are presented as mean ± standard deviation for normally distributed variables (Age, Leukocytes, Neutrophil; Shapiro–Wilk *p* > 0.05) and median (interquartile range [Q1–Q3]) for non-normally distributed variables. IQR: interquartile range; PLR: platelet-to-lymphocyte ratio; NLR: neutrophil-to-lymphocyte ratio; SII: systemic immune-inflammation index; GLR: glucose-to-lymphocyte ratio; LVSI: lymphovascular space invasion. * indicates statistical significance (*p* < 0.05).

**Table 2:** Multivariable logistic regression for the prediction of lymphovascular space invasion (LVSI) using continuous predictors.

Variable	β (SE)	Adjusted OR (95% CI)	*p *-Value	VIF
Age (per 1-year increase)	−0.032 (0.030)	0.968 (0.912–1.027)	0.286	1.00
Tumor size (per 1 cm increase)	0.467 (0.140)	**1.595 (1.211–2.099)**	**0.001**	1.05
PLR (per 1-unit increase)	0.013 (0.006)	**1.013 (1.001–1.024)**	**0.033**	1.06
Intercept	−4.013 (1.985)	—	0.043	—

Bold *p*-values indicate statistical significance (*p* < 0.05). Abbreviations: β, regression coefficient; SE, standard error; OR, odds ratio; CI, confidence interval; PLR, platelet-to-lymphocyte ratio; VIF, variance inflation factor; LVSI, lymphovascular space invasion.

## Data Availability

The raw data supporting the conclusions of this article will be made available by the authors on request.

## Abbreviations

AUC	Area Under the Curve
BSO	Bilateral Salpingo-Oophorectomy
CA-125	Cancer Antigen 125
CI	Confidence Interval
COVID-19	Coronavirus Disease 2019
DCA	Decision Curve Analysis
EC	Endometrial Cancer
EPV	Events Per Variable
FIGO	International Federation of Gynecology and Obstetrics
GLR	Glucose-to-Lymphocyte Ratio
HR	Hazard Ratio
IQR	Interquartile Range
LVSI	Lymphovascular Space Invasion
MRI	Magnetic Resonance Imaging
NLR	Neutrophil-to-Lymphocyte Ratio
NSMP	Non-specific Molecular Profile
OR	Odds Ratio
PDGF	Platelet-Derived Growth Factor
PLR	Platelet-to-Lymphocyte Ratio
POLE	DNA Polymerase Epsilon
ROC	Receiver Operating Characteristic
SE	Standard Error
SII	Systemic Immune-Inflammation Index
TAH	Total Abdominal Hysterectomy
TGF-β	Transforming Growth Factor-Beta
VEGF	Vascular Endothelial Growth Factor
VIF	Variance Inflation Factor
